# Differentiation and Recruitment of Th9 Cells Stimulated by Pleural Mesothelial Cells in Human *Mycobacterium tuberculosis* Infection

**DOI:** 10.1371/journal.pone.0031710

**Published:** 2012-02-20

**Authors:** Zhi-Jian Ye, Ming-Li Yuan, Qiong Zhou, Rong-Hui Du, Wei-Bing Yang, Xian-Zhi Xiong, Jian-Chu Zhang, Cong Wu, Shou-Ming Qin, Huan-Zhong Shi

**Affiliations:** 1 Key Laboratory of Pulmonary Diseases of Health Ministry, Department of Respiratory Diseases, Union Hospital, Tongji Medical College, Huazhong University of Science and Technology, Wuhan, China; 2 Department of Internal Medicine, Wuhan Institute of Tuberculosis Prevention and Control, Wuhan, China; 3 Institute of Respiratory Diseases, First Affiliated Hospital, Guangxi Medical University, Nanning, China; University of Cape Town, South Africa

## Abstract

Newly discovered IL-9–producing CD4^+^ helper T cells (Th9 cells) have been reported to contribute to tissue inflammation and immune responses, however, differentiation and immune regulation of Th9 cells in tuberculosis remain unknown. In the present study, our data showed that increased Th9 cells with the phenotype of effector memory cells were found to be in tuberculous pleural effusion as compared with blood. TGF-β was essential for Th9 cell differentiation from naïve CD4^+^ T cells stimulated with PMA and ionomycin *in vitro* for 5 h, and addition of IL-1β, IL-4 or IL-6 further augmented Th9 cell differentiation. Tuberculous pleural effusion and supernatants of cultured pleural mesothelial cells were chemotactic for Th9 cells, and this activity was partly blocked by anti-CCL20 antibody. IL-9 promoted the pleural mesothelial cell repairing and inhibited IFN-γ-induced pleural mesothelial cell apoptosis. Moreover, pleural mesothelial cells promoted Th9 cell differentiation by presenting antigen. Collectively, these data provide new information concerning Th9 cells, in particular the collaborative immune regulation between Th9 cells and pleural mesothelial cells in human *M. tuberculosis* infection. In particular, pleural mesothelial cells were able to function as antigen-presenting cells to stimulate Th9 cell differentiation.

## Introduction

Tuberculosis presents a challenging worldwide public heath problem. According to the World Health Organization, one third of the world's population are thought to be infected with *M. tuberculosis*, but only 10% of the infected individuals would develop active tuberculosis [1]. Most infected individuals will stay healthy throughout their lifetime and develop a latent infection with no sign of disease. They can be regarded as being protected against the disease by the immune response induced through natural infection. Infection with *M. tuberculosis* elicits humoral and cellular immune responses, and T cell-mediated immunity, comprising CD4^+^ and CD8^+^ cells, is thought to be important for effective prevention of disease after *M. tuberculosis* infection [2]. *M. tuberculosis* are seldom eradicated, however, and a few *M. tuberculosis* can persist for years, residing inside macrophages in granulomas and evading elimination by the host immune response [3].

Tuberculous pleural effusion (TPE) is caused by a severe delayed-type hypersensitivity reaction in response to the rupture of a subpleural focus of *M. tuberculosis* infection. An accumulation of lymphocytes, especially CD4^+^ T cells, in TPE has been well documented [4]. Actually, more and more data have demonstrated that several Th subsets, such as Th1 cells [5], Th17 cells [6], and regulatory T cells [7], etc. are involved in the pathogenesis of TPE.

The cytokine IL-9 was identified, and its basic features were described more than two decades ago [8], [9]. IL-9 has long been thought to be a Th2 cytokine, as it promotes allergic inflammation and is associated with various Th2 responses [10]. More recent studies revealed the multifunction activities of this cytokine. Of significant importance is the recent discovery of a Th subset of IL-9–producing CD4^+^ T cells (Th9 cells) distinct from Th1, Th2, or Th17 cells [11], [12]. Th9 cells are characterized by production of IL-9 and IL-10 and develop from naïve CD4^+^ precursors driven by the combined effects of TGF-β and IL-4 [11], [12]. Th9 cells have been reported to be capable of inducing tissue inflammation in a colitis model [13]; however, whether Th9 cells are involved in infection immunity, especially in *M. tuberculosis* infection, have not been investigated.

Pleural mesothelial cells (PMCs), presented in a single layer covering each pleural membrane, are exposed to a microenvironment with high levels of cytokines and chemokines during infection [14]. Early studies have demonstrated that PMCs facilitate monocyte transmigration across pleural mesothelium during *M. tuberculosis* infection [15]. In the present study, we investigated the distribution of Th9 cells in TPE, the phenotypic characteristics of Th9 cells, the possible mechanisms of differentiation and recruitment of Th9 cells into pleural space, and the capabilities of PMCs to stimulate Th9 cell differentiation in response to *M. tuberculosis* antigens.

## Results

### Increased proportions of Th9 cells in TPE

**Figure 1:**
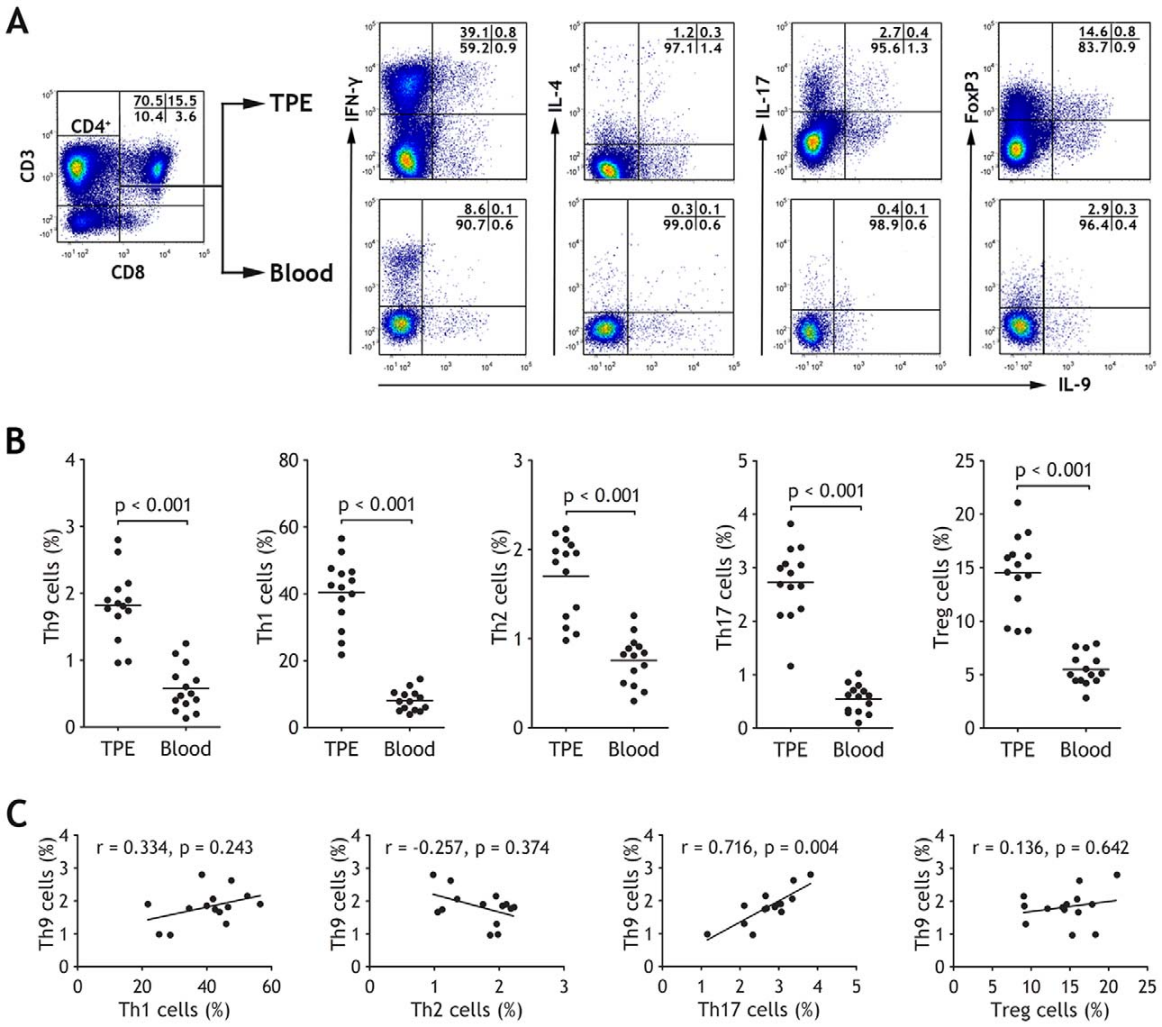
Th9 cells increased in tuberculous pleural effusion (TPE). (A) Th9 cells within CD4^+^ T cells were identified based on their expression of CD3 and not of CD8. The representative flow cytometric dot-plots of Th9, Th1, Th2 cells, Th17, and Tregs in TPE and blood are shown. (B) Comparisons of percentages of Th9, Th1, Th2 cells, Th17, and Tregs in TPE and blood (n = 14). Horizontal bars indicate means. The percentages of Th cells represented Th cell numbers in total CD4^+^ T cell numbers as determined by flow cytometry, comparison was made using a Wilcoxon signed-rank test. The percentages of Th cells were determined by flow cytometry, comparison was made using a Wilcoxon signed-rank test. (C) Th9 cells correlated negatively with Th1, Th2 cells, Th17, and Tregs in TPE (n = 14). Correlations were determined by Spearman rank correlation coefficients.

We first investigated the distribution of Th9 cells in relation to Th1, Th2, Th17 cells and regulatory T cells (Tregs) in TPE. Flow cytometry was performed on mononuclear cells from TPE and peripheral blood with gating on CD3^+^ and CD8^−^ T cells (Figure 1A). We noted that IL-9-producing CD4^+^ T cells presented in TPE and blood. Unlike murine Th9 cells [12], human Th9 cells did not express IL-10 (data not shown). In addition, we also observed some Th9/Th1 and Th9/Treg, to a less extent, Th9/Th2 and Th9/Th17 cells in TPE or blood (Figure 1A).

As shown in Table 1, we noted that the numbers Th cells in TPE and blood with *M. tuberculosis*-specific stimulation were very low, it would not be possible for us to investigate the phenotypic characteristics of Th9 cells, we thus used PMA and ionomycin to stimulate naïve CD4^+^ T cells *in vitro* for 5 h throughout the whole study.

**Table 1:** Comparisons of Th cells in tuberculous pleural effusion (TPE) and blood stimulated with PMA+ionomycin or tuberculosis antigens*.

	PMA+ionomycin		Tuberculosis antigens	
	TPE	Blood	TPE	Blood
Th9 cells (%).	1.82±0.14† ‡	0.58±0.09‡	0.31±0.02†	0.10±0.01
Th1 cells (%)	40.44±2.67† ‡	8.05±0.85‡	2.06±0.09†	0.52±0.03
Th2 cells (%)	1.70±0.12† ‡	0.76±0.07‡	0.26±0.03†	0.07±0.01
Th17 cells (%)	2.73±0.18† ‡	0.55±0.07‡	0.46±0.01†	0.09±0.01

*Values are presented as mean ± SEM, n = 14.

† p<0.01 compared with the corresponding blood with the same stimulation determined by Wilcoxon signed-rank test.

‡ p<0.01 compared with the corresponding compartments stimulated by tuberculosis antigens determined by Wilcoxon signed-rank test.

As shown in Figure 1B, percentages of Th9 cells represented the higher values in TPE (1.82±0.14%), showing a significant increase in comparison with those in the corresponding blood (0.58±0.09%, Wilcoxon signed-rank test, n = 14, p<0.001). Similar increases were observed in pleural Th1, Th2, Th17 cells and Tregs (40.44±2.67%, 1.70±0.12%, 2.73±0.18% and 14.53±0.96%, respectively), compared with their corresponding compartments in blood (8.05±0.85%, 0.76±0.07%, 0.55±0.07% and 5.48±0.40%, respectively) (all p<0.001).

Since previous studies reported that IL-9 was also secreted by Th17 cells [16], [17] or Tregs [18], and that Th9 cells were tightly associated with Th2 cells [11], we therefore explored the correlationship between Th9 cells and the other subsets. Our data showed that the numbers of Th9 cells were positively correlated with the numbers of Th17 (r = 0.716, p = 0.004), but not with Th1 (r = 0.334, p = 0.243), Th2 cells (r = −0.257, p = 0.374), or Tregs (r = 0.136, p = 0.642) (Figure 1C).

### Phenotypic characteristics of Th9 cells

**Figure 2:**
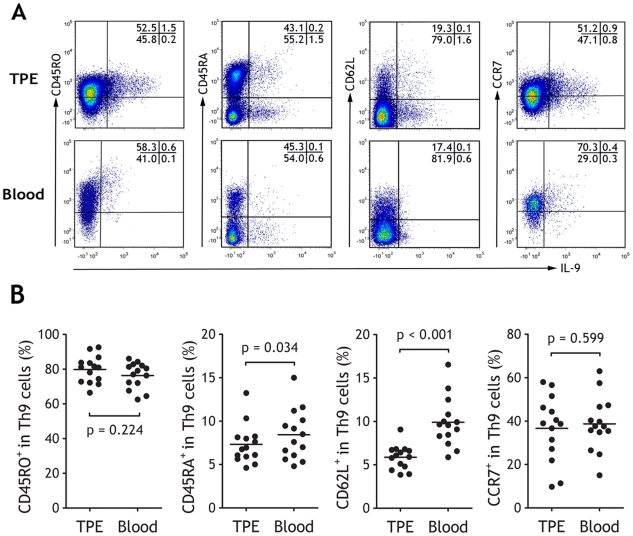
Phenotypic characteristics of Th9 in tuberculous pleural effusion (TPE). (A) The representative dot plots showing expressions of IL-9 and CD45RO, CD45RA, CD62L, or CCR7 on CD4^+^ T cells. (B) Comparisons of percentages of CD45RO^+^, CD45RA^+^, CD62L^+^, CCR7^+^ cells in total Th9 cells in TPE and blood from patients with TPE (n = 14). The data are calculated by dividing the numbers in upper right quadrants by the numbers in both upper and lower right quadrants. Horizontal bars indicate means; comparison was made using a Wilcoxon signed-rank test.

We observed that most Th9 cells expressed high level of CD45RO in both TPE and blood (79.80±2.07% versus 76.30±1.99%, n = 14, Wilcoxon signed-rank test, p = 0.224), low levels of CD45RA (7.33±0.61% versus 8.43±0.77%, p = 0.034), and low levels of CD62L (5.88±0.37% versus 9.89±0.76%, p<0.001), indicating that they were memory cells, especially those in TPE (Figure 2). Also as shown in Figure 2, pleural and blood Th9 cells expressed certain level of CCR7 (36.66±4.03% versus 38.72±3.34%, p = 0.599).

**Figure 3:**
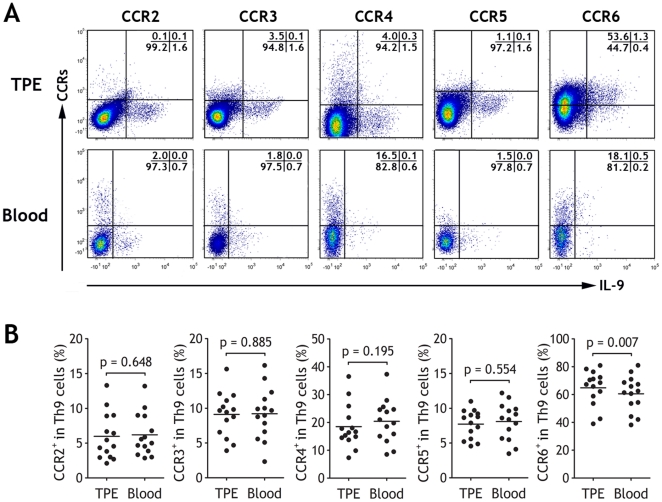
Chemokine receptors expressed on Th9 cells. (A) Flow cytometric dot-plots of expressions of CCR2, CCR3, CCR4, CCR5, and CCR6 on Th9 cells from tuberculous pleural effusion (TPE) and blood. (B) Comparisons of percentages of CCR2^+^, CCR3^+^, CCR4^+^, CCR5^+^, and CCR6^+^ cells in total Th9 cells in TPE and blood from patients with TPE (n = 14). The data are calculated by dividing the numbers in upper right quadrants by the numbers in both upper and lower right quadrants. Horizontal bars indicate means; comparisons of CCR expressions were made using a Wilcoxon signed-rank test.

Except for CCR7, the expression profiles of the other CCRs studied on Th9 cells are shown in Figure 3. Overall, Th9 cells in both TPE and blood expressed low levels of CCR2, CCR3 and CCR4 and CCR5, and there were no differences between TPE and blood (all p>0.05). We noted that most pleural Th9 cells were positive for CCR6 (64.82±3.36%), which were higher than blood Th9 cells (60.53±3.41%, p = 0.007).

### Contribution of Cytokines to Differentiation of Th9 cells

**Figure 4:**
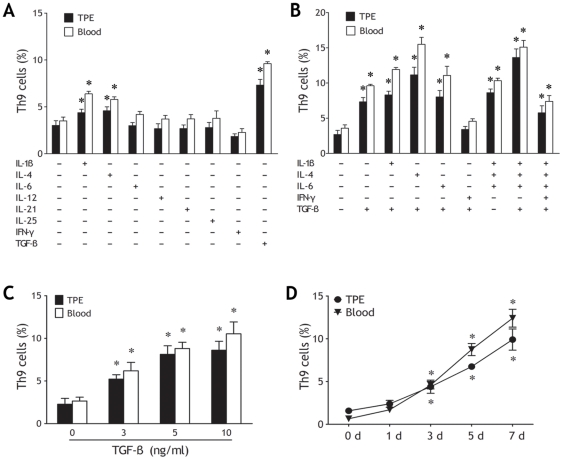
Differentiation of human Th9 cells from naïve CD4^+^ T cells stimulated by different cytokines. Purified naïve CD4^+^ T cells isolated from tuberculous pleural effusion (TPE) and blood (both n = 5) were stimulated with plate-bound anti-CD3 and soluble anti-CD28 mAbs in the presence of the indicated cytokines, either alone (A) or in various combinations (B). Seven days after activation, the cells were stimulated with PMA and ionomycin for 5 h and analyzed for IL-9 expression after intracellular staining. The comparisons were determined by Kruskal-Wallis one-way analysis of variance on ranks. * p<0.05 compared with medium control. Naïve CD4^+^ T cells from TPE and blood (both n = 5) were cultured and stimulated with indicated concentrations of TGF-β for 7 d (C), or with 5 ng/ml of TGF-β for indicated time points (D), the percentages of Th9 cells determined by flow cytometry. Comparisons were determined by Kruskal-Wallis one-way analysis of variance on ranks. * p<0.05 compared with baseline values.

We purified CD4^+^ T cells and cultured them *ex vivo* with plate-bound anti-CD3 and soluble anti-CD28 mAbs in the presence of one or more of IL-1β, IL-4, IL-6, IL-12, IL-21, IL-25, IFN-γ and TGF-β for 7 d. With IL-2–containing medium provided a baseline for comparison, IL-1β, IL-4 and TGF-β alone could promote the differentiation of Th9 cells from CD4^+^ T cells, and the most significant effect was seen with TGF-β (Figure 4A). On the contrary, IFN-γ alone slightly suppressed Th9 cell differentiation (Figure 4A). As expected, the addition of IL-1β, IL-4, IL-6, or their combination enhanced TGF-β induced Th9 cell differentiation, with TGF-β plus IL-4 eliciting the most amount of Th9 cells (Figure 4B). Additionally, IFN-γ exhibited a strong suppressive capacity on Th9 cell differentiation induced by these cytokines.

Since TGF-β showed a potent capability to induce Th9 cell differentiation, we further examined impact of TGF-β on kinetics of Th9 differentiation, and found that the production of IL-9 was induced by TGF-β in a dose-dependent and time-dependent manner (Figure 4C and D).

We also noted that above mentioned proinflammatory cytokines or their combinations could promote differentiation of Th9 cells from blood naïve CD4^+^ T cells in a similar manner (Figure 4).

### Recruitment of Th9 cells into TPE might be induced by chemokine CCL20

**Figure 5:**
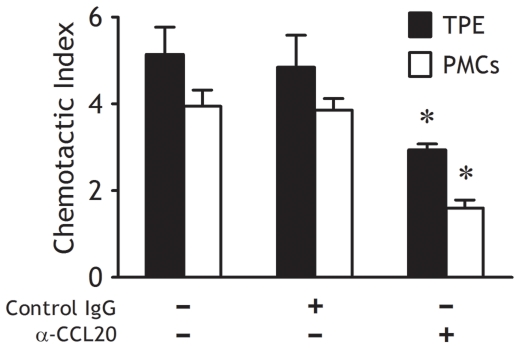
Chemokine CCL20 in tuberculous pleural effusion (TPE) was chemotactic for Th9 cells *in vitro*. TPE and supernatants of cultured pleural mesothelial cells (both n = 5) were used to stimulate chemotaxis of Th9 cells in the absence of presence of anti–CCL20 mAb or an irrelevant isotype control. The comparisons were determined by Kruskal-Wallis one-way analysis of variance on ranks. *p<0.05 compared with the irrelevant isotype control.

It was found that concentration of CCL20 in TPE was much higher than that in serum, and that significant expression of CCL20 was observed in all PMCs (our unpublished data). These data suggested that PMCs might be the cell sources of the elevated pleural CCL20. Taken together with our observation in the present study that Th9 cells expressed relative high level of CCR6 (Figure 3), which is a ligand for CCL20, we hypothesized that Th9 cells could migrate into the pleural space in response to CCL20. Indeed, both TPE and supernatants of cultured PMCs exerted a potent chemoattractant activity for circulating Th9 cells, an anti-CCL20 mAb significantly suppressed Th9 cell chemotaxis (Figure 5). Therefore, recruitment of Th9 cells into TPE might be induced by PMCs via CCL20-CCR6 axis.

### Effects of IL-9 on wound healing of PMCs

**Figure 6:**
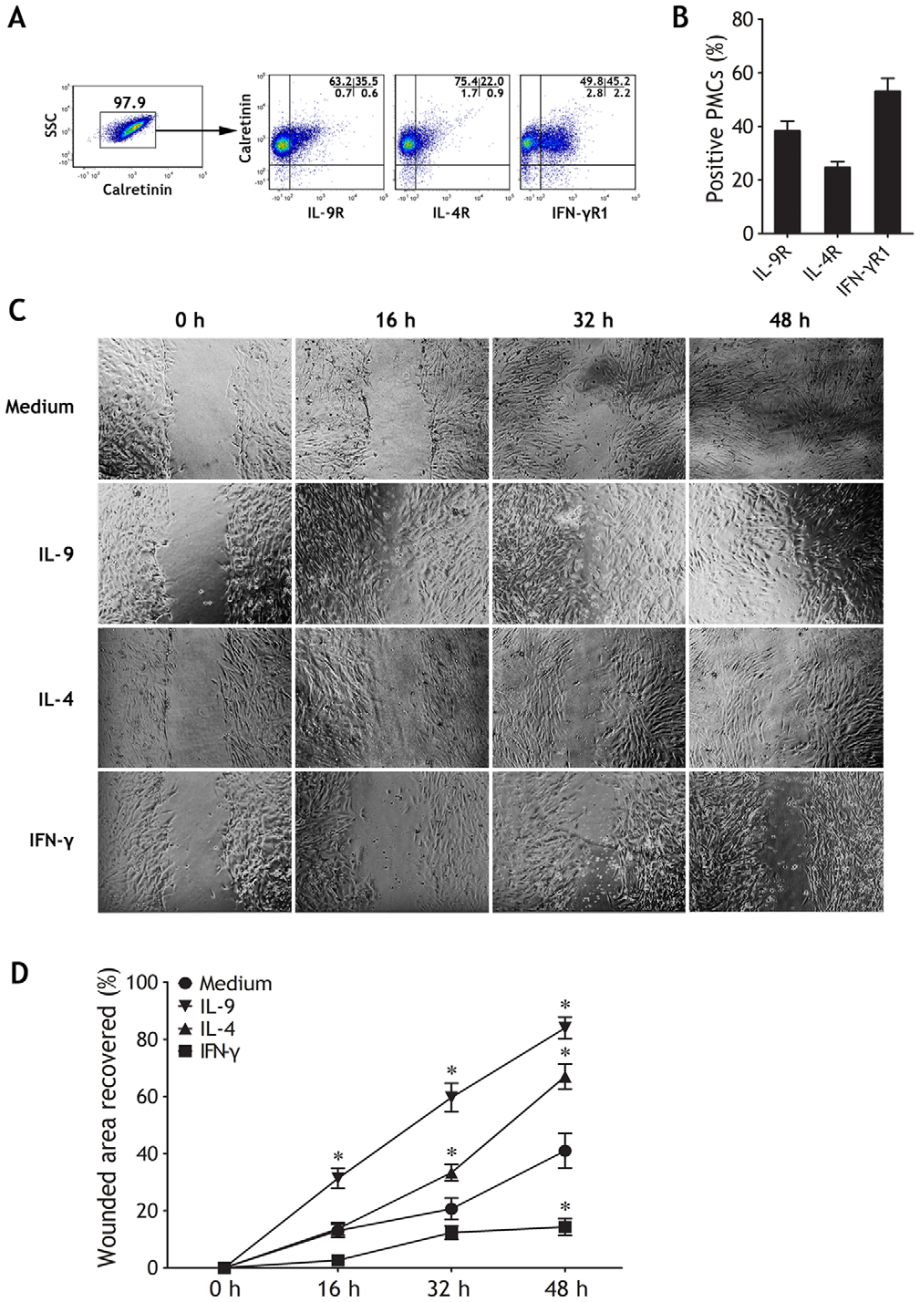
Effects of IL-9, IL-4 and IFN-γ on wound healing of *in vitro* injury model of pleural mesothelial cells. (A) Pleural mesothelial cells was identified by expression of calretinin and sideward scatter (SSC) using flow cytometry, the representative flow cytometric dot-plots are showing expression of IL-9R, IL-4R, and IFN-γR1 on PMCs from tuberculous pleural effusion. (B) Summary dada of percentages of IL-9R^+^, IL-4R^+^, and IFN-γR1^+^ PMCs (n = 5). (C) Microscopic photography after wound induction on a confluent monolayer of PMCs in a time course from 16 to 48 h revealed that wound healing was enhanced by IL-9 or IL-4 and retarded by IFN-γ (Original magnification: ×200). (D) Graphs show relative wound closure over time, based on the wound gap compared with initial wound size. Mean ± SEM of 5 independent experiments. The comparisons were determined by Kruskal-Wallis one-way analysis of variance on ranks, *p<0.05 compared with medium control at the same time points.

As shown in Figure 6A and B, substantial expressions of IL-9 receptor (IL-9R, 38.36±3.63%, n = 5), IL-4R (24.68±2.13%), and IFN-γR (53.14±4.82%) were observed on PMCs isolated from TPE. We thus sought to investigate whether IL-9, IL-4, or IFN-γ were involved in the regulation of mesothelial membrane repairing. To evaluate effects of these cytokines on growth of PMCs in early stage of repairing, an *in vitro* injury model was used. As soon as 16 h after wounding, both IL-9 and IL-4 had substantial and persistent improving effects on PMC layer closure. Unexpectedly, IFN-γ was noted to be harmful to PMC wound healing during the whole 48 h-culture (Figure 6C and D).

**Figure 7:**
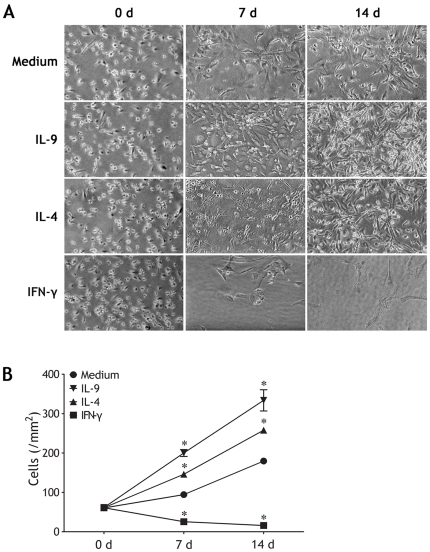
Effects of IL-9, IL-4 and IFN-γ on long-term restoring of pleural mesothelial cells (PMCs). (A) Pleural mesothelial cells were seeded in Petri dishes in complete medium in the presence of IL-9, IL-4 or IFN-γ for 14 d to allow for their growth. Representative of 5 independent experiments (Original magnification: ×400). (B) Comparisons of PMC numbers in each group (n = 5). The comparisons were determined by Kruskal-Wallis one-way analysis of variance on ranks, *p<0.05 compared with medium control at the same time points.

Besides early stage of repairing, long term restoring might further reflect the local remodeling of mesothelial wounded area. In *in vitro* experiments of long term culture of PMCs designed for evaluating effects of the above cytokines on growth of PMCs in late stage of repairing, both IL-9 and IL-4 could significantly improve long term restoring of PMCs represented by the density of cells; in contrast, IFN-γ even severely impaired this restoring (Figure 7).

### Apoptosis of PMCs was induced by IFN-γ and inhibited by IL-9 and IL-4

**Figure 8:**
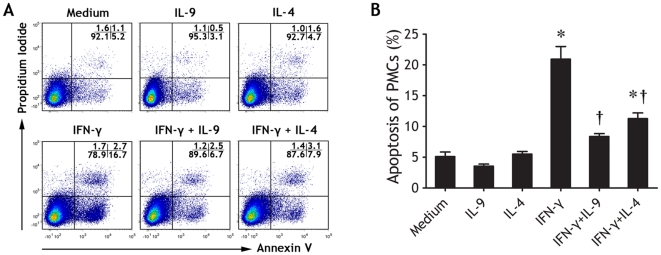
Effects of IL-9, IL-4 and IFN-γ on apoptosis of pleural mesothelial cells (PMCs). PMCs were seeded in Petri dishes in complete medium in the presence of one or more of IL-9, IL-4, and IFN-γ for 48 h. (A) The representative flow cytometric dot-plots are showing Annexin V/propidium iodide co-staining for identification of apoptotic PMCs. (B) Comparisons of apoptotic PMCs in each group (n = 5). The comparisons were determined by Kruskal-Wallis one-way analysis of variance on ranks, *p<0.05 compared with medium control, †p<0.05 compared with IFN-γ alone.

Apoptosis is an essential form of cell death important in development, in physiological cell turnover and in deletion of damaged cells [19]. Under normal circumstances, PMC population turns over slowly, with proliferation balanced by cell death. Exposure of PMCs to injury or inflammation disrupts this balance. Little is known about the role of apoptosis in PMC biology. Most of the studies that have addressed this issue have focused on the mesothelial response to asbestos [20]. Together with the negative effect of IFN-γ in PMC wound healing and long term restoring as above mentioned, we hypothesized that IFN-γ might be responsible for the cell death of PMCs. Indeed, neither IL-9 nor IL-4 affected apoptosis of PMCs, whereas IFN-γ could induce significantly PMC apoptosis. We further noted that both IL-9 and IL-4 inhibited IFN-γ-induced PMC apoptosis (Figure 8).

### Antigen presentation to CD4^+^ T cells by PMCs *in vitro*

**Figure 9:**
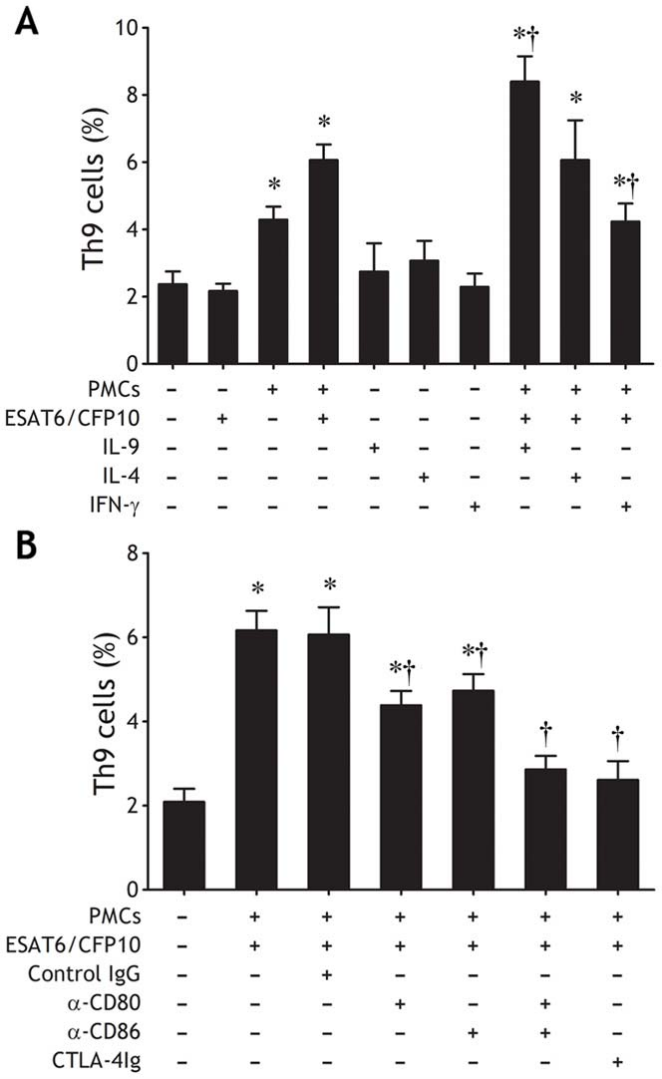
*In vitro* stimulation of Th9 cell differentiation by antigen presentation of pleural mesothelial cells (PMCs). Purified naïve CD4^+^ T cells from blood were cultured with autologous PMCs at a ratio of 5 ∶ 1 for 5 d in the absence or presence of exogenous antigen ESAT-6/CFP-10, IL-9, IL-4 or IFN-γ (A), or anti–CD80, –CD86 mAb, a combination of anti–CD80 and –CD86 mAbs, CTLA-4Ig or control Ig (B) was added into the coculture, the frequencies of Th9 cells were determined by flow cytometry. The results are reported as mean ± SEM from 5 independent experiments. The comparisons were determined by Kruskal-Wallis one-way analysis of variance on ranks. * p<0.05 compared with medium control, †p<0.05 compared with PMCs plus ESAT-6/CFP-10 (or plus control IgG).

We have found that Class II MHC protein, HLA-DR, was well expressed on PMCs, and that these PMCs also expressed high levels of CD80 and CD86, two proteins with important roles as co-stimulatory signals for T cell responses (our unpublished data) [21]. To assess the capacity of PMCs to function as antigen-presenting cells to stimulate Th9 cell differentiation, purified naïve CD4^+^ T cells from blood were cultured alone or with PMCs at a ratio of 5 ∶ 1 for 5 d. We noted that in the absence of PMCs, the numbers of Th9 cells were quite low even in the presence of 10 µg/ml of exogenous *M. tuberculosis*-specific peptides of early secretory antigenic target-6 kDa/culture filtrate protein-10 (ESAT-6/CFP-10); the addition of PMCs yielded an significant increase in Th9 cells even in the absence of ESAT-6/CFP-10, compatible with PMCs presenting processed endogenous antigen to which they were might expose within TPE environment to CD4^+^ T cells. The addition of exogenous ESAT-6/CFP-10 to the PMC-CD4^+^ T cell co-culture resulted in even greater Th9 cell differentiation indicative that PMCs *in vitro* were capable of processing antigen (Figure 9A). We also noted that IL-9 but not IL-4, significantly enhanced Th9 cell differentiation induced by antigen presentation of PMCs. In contrast, IFN-γ inhibited its differentiation (Figure 9A).

To further evaluate whether PMCs were providing requisite B7 costimulatory signals for their antigen-presenting function [21], we assessed the roles of CD80 and CD86 as co-stimulatory signals in PMC antigen presentation to CD4^+^ T cells *in vitro.* We cultured CD4^+^ T cells and PMCs with ESAT-6/CFP-10 in the presence or absence of inhibitory concentrations of anti-CD80 mAb, anti-CD86 mAb, a combination of both, or a soluble fusion protein of extracellular domain of cytotoxic T lymphocyte-associated antigen-4 and the Fc portion of IgG (CTLA-4Ig). Anti-CD80 and anti-CD86 mAb alone partially blocked Th9 cell differentiation; a combination of both anti-CD80 and anti-CD86 blocking mAbs and CTLA-4Ig yielded even greater inhibition of PMC-elicited Th9 cell differentiation (Figure 9B).

## Discussion

Since the identification of Th1/Th2 cells more than two decades ago, followed by Tregs and Th17 cells, and now Th9 cells have been added to the ‘portfolio’ of Th cells. Although Th9 cells have been described in mice [11], [12], they are not very well characterized in human; especially, no data concerning whether Th9 cells are involved in infection are available so far. Although some studies have demonstrated that Th9 might elicit inflammation [12] and contribute to the development of allergic diseases [22], [23], the role of this new Th subset in immune response remains to be further elucidated. To the best of our knowledge, the present study was the first one investigating the role of Th9 cells in human tuberculosis.

In the present study, we have demonstrated that Th9 cells were present in TPE, and the numbers of Th9 cells represented in TPE were much higher than those in the corresponding blood. Majority of these pleural Th9 cells displayed the phenotype of effector memory cells, since they expressed high levels of CD45RO, very low levels of CD62L and relative low levels of CCR7. The molecular mechanisms underlying the generation and differentiation of human Th9 cells are not elucidated completely. Studies in mice have shown that TGF-β derived Th2 cells to lose their characteristic profile and switched to Th9 cells or, in combination with IL-4, promoted the differentiation of Th9 cells directly [11], and that IL-4 blocked the generation of TGF-β-induced Tregs and instead induced the development of Th9 cells that could also produced IL-10 [12]. It has been reported that the transcription factor PU.1 and IFN-regulatory factor 4 were required for the development of murine Th9 cells [22], [23]. In human, memory CD4^+^ T cells have been reported to be induced to become Th9 cells [24]. Similar to the findings in mouse studies, TGF-β and IL-4 induced the differentiation of human Th9 cells *in vitro*; and IL-1β, IL-6, IL-10, IFN-α, IFN-β or IL-21 could augment Th9 differentiation, while IFN-γ and IL-27 partially suppressed Th9 differentiation [25]. Our study draw the similar conclusion, showing that TGF-β was essential for Th9 differentiation from naïve CD4^+^ T cells isolated from TPE or blood, addition of IL-4, IL-1β, or IL-6 augmented IL-9 production, and the production of IL-9 induced by TGF-β was in a dose- and time-dependent manner. Consistent with Wong et al. [25], we showed that IFN-γ significantly suppressed IL-9 production induced by TGF-β. The fact that although the concentration of IFN-γ in TPE was much higher than that in serum [26], the numbers of Th9 cells were higher in TPE than in blood, indicated that there was complex cytokine network in the regulation of Th9 cell differentiation in TPE, and there might exist other mechanisms besides local differentiation leading to Th9 cells increase in TPE.

On the other hand, an increase in numbers of Th9 cells in TPE might also be due to Th9 cell recruitment from peripheral blood. In the previous study, we provided direct evidence that IL-16 is capable of inducing CD4^+^ T cell infiltration into the pleural space [27]. Therefore, as a subpopulation of CD4^+^ T cells, Th9 cells might also be recruited in to TPE by local production of IL-16, since IL-16 level is significantly higher in TPE than in serum [27]. In the present study, we were prompted to evaluate whether chemokine/CCR axis was responsible for the influx of Th9 cells. It was found that all PMCs from TPE expressed CCL20, and that CCL20 concentration in TPE was much higher than those in serum (our unpublished data), and that Th9 cells in both TPE and blood expressed high level of CCR6 on their surface. These data suggested that CCL20/CCR6 axis might be related to the accumulation of Th9 cells in TPE. Indeed, an *in vitro* migration assay further confirmed that both TPE and supernatants of cultured PMCs could induce the migration of Th9 cells, and that anti–CCL20 mAb significantly inhibited the ability of TPE or supernatants to stimulate Th9 cell chemotaxis. Therefore, PMC-produced CCL20 might be able to chemoattract Th9 cell recruitment into pleural space during *M. tuberculosis* infection.

Our data also showed that the numbers of Th9 cells in TPE positively correlated with that of Th17 cells, but not of Th1, Th2, or Tregs. We supposed the finding that IL-9 together with TGF-β promoted Th17 differentiation from naïve CD4^+^ T cells [28] might account for this correlation. In addition, Zhou et al. [29] has reported that IL-9 promoted Th17 cell migration into the central nervous system via CCL20 produced by astrocytes, and we speculated that IL-9 may also contribute to Th17 migration into TPE, which need to be further investigated.

The pathophysiological functions of Th9 cells in *M. tuberculosis* infection have not been investigated. Findings from one *in vitro* study suggested that increased expression of IL-9 may contribute to the development of tuberculosis [30]. Since PMCs are an important component of the pleural environment, they may collaborate with the other kinds of cells, including Th9 cells, in the generation of local cell-mediated immunity to various pathogens, including *M. tuberculosis*. The mesothelium is a slowly renewing tissue that can be stimulated by a variety of agents and direct physical damage to increase its turnover rate [31]. We investigated whether Th9, Th2, or Th1 cells were involved in the regulation of mesothelial membrane repairing. Indeed, our *in vitro* experiments showed that both IL-9 and IL-4 significantly improved wound healing and long term restoring of PMCs; in contrast, IFN-γ even severely impaired this wound healing and restoring.

It has become clear that both cell proliferation and apoptosis are highly regulated processes within the cell with very specific signals regulating the stepwise processes. Apoptosis is genetically programmed cell death triggered by external stimuli, and is an important process for normal tissue development and homeostasis [20]. An understanding of the balance between proliferation and apoptosis in PMCs exposed to environmental inflammation is critical to further understanding of the mechanisms and patterns of pleural injury and fibrogenesis, which occur frequently during *M. tuberculosis* infection [32]. Our data showed as Th1 cell-derived cytokine, IFN-γ not only impaired PMC wound healing but also induced PMC apoptosis, we hypothesized that IFN-γ might be responsible for the cell death of PMCs. In contrast, neither IL-9 nor IL-4 affected apoptosis of PMCs, both cytokines further inhibited IFN-γ-induced PMC apoptosis.

In the present study, we were also interested in knowing whether PMCs could promote differentiation of pleural Th9 cells in *M. tuberculosis* infection. We found in the present study that in the coculture with purified naïve CD4^+^ T cells, PMCs promoted significantly differentiation of Th9 cells could be observed even in the absence of exogenous *M. tuberculosis*-specific peptides of ESAT-6/CFP-10; the addition of exogenous ESAT-6/CFP-10 yielded a more intensive differentiation of Th9 cells in CD80- and CD86-dependent means. These data suggested that exposures of PMCs to *M. tuberculosis*-related antigen was shown to be sufficient for these PMCs serving as antigen-presenting cells to present antigens to CD4^+^ T cells in *in vitro* assays, and that PMCs could further process exogenous antigen during *in vitro* culture.

Additionally, we found that IL-9 intensively amplified Th9 cell differentiation induced by antigen presentation of PMCs. We reasoned this amplification might result from that IL-9 prevented PMCs from apoptosis and improved wound healing along with long term restoring of PMCs. As expected, IFN-γ inhibited Th9 cell differentiation induced by antigen presentation of PMCs due to that it impaired PMC wound healing and induced PMC apoptosis. Interestingly, although IL-4 also showed the impacts of anti-apoptosis on PMCs, it did not amplify Th9 cell differentiation induced by antigen presentation of PMCs. Since our study suggested that specific accessory molecules played an important role in PMCs antigen presentation, we wondered whether IL-9 or IL-4 affected PMCs antigen presentation function by regulating these co-stimulatory molecules expression (our ongoing study).

An obvious limitation of this study was that we did not know the exact duration of TPE, since the development of TPE had occurred before the patients' hospitalization. It should be interesting to elucidate whether collecting pleural fluid at the starting or the resolution point of the disease would affect the functional findings of Th9 cells observed in the current study. It should also be noted that Th9 cells from patients with an earlier stage of TPE might show a different phenotype compared to those from patients with advanced/progressed stages of the disease. However, there was no way to classify patients with TPE into early and advanced/progressed stages so far.

Another important issue was that for the Th9 phenotypic studies, intra- and surface markers were measured in T-cells after stimulation with PMA and ionomycin, but not with tuberculosis-specific antigen, although the polyclonal stimulation with PMA and ionomycin could not represent *M. tuberculosis*-specific immune responses, and might also increase the expression levels of all markers. Our current data confirmed that Th9 cell numbers in TPE and blood with tuberculosis-specific stimulation were very low, they would not be sufficient for investigating the phenotypic characteristics of Th9 cells. We therefore used PMA plus ionomycin to stimulate naïve CD4^+^ T cells *in vitro* for 5 h.

In conclusion, our data showed that the numbers of Th9 cells with the phenotype of effector memory cells in TPE were significantly increased when compared with their compartments in blood, and that overrepresentation of Th9 cells in TPE may be due to the increased local proinflammatory cytokines and to PMC-produced CCL20. Our data also showed that IL-9 significantly improved PMC wound healing and long term restoring and inhibited IFN-γ-induced PMC apoptosis. Moreover, PMCs were able to function as antigen-presenting cells to stimulate Th9 cell differentiation in response to *M. tuberculosis* antigens.

## Materials and Methods

### Subjects

The study protocol was approved by our institutional review boards for human studies, and informed consent was obtained from all subjects. Fourteen patients (age range: 21 to 64 yr) were proven to have TPE, as evidenced by growth of *M. tuberculosis* from pleural fluid or by demonstration of granulomatous pleurisy on closed pleural biopsy specimen in the absence of any evidence of other granulomatous diseases. All TPE patients were anti-human immunodeficiency virus antibody negative and were recruited from Department of Internal Medicine, Wuhan Institute of Tuberculosis Prevention and Control. After anti-tuberculosis chemotherapy, the resolution of TPE and clinical symptoms was observed in all patients.

The patients were excluded if they had accepted any invasive procedures directed into the pleural cavity or if any chest trauma was occurred within 3 months prior to their hospitalization, or if the existence of a pleural effusion of origin unknown. At the time of sample collection, none of the patients had received any anti-tuberculosis therapy, corticosteroids, or other nonsteroid anti-inflammatory drugs.

### Sample collection and processing

Five-hundred to 1,000 ml of TPE samples from each patient were collected in heparin-treated tubes, through a standard thoracocentesis technique within 24 h after hospitalization. Twenty milliliters of blood were drawn simultaneously. TPE specimens were immersed in ice immediately and were then centrifuged at 1,200 g for 5 min. The cell pellets of TPE were resuspended in HBSS, and mononuclear cells were isolated by Ficoll-Hypaque gradient centrifugation (Pharmacia, Uppsala, Sweden) to determine the T cell subsets within 1 h. A pleural biopsy was performed when the results of pleural fluid analysis were suggestive of tuberculosis.

### Flow cytometry

The expression of markers on T cells from TPE and blood were determined by flow cytometry as previously described [33] after surface or intracellular staining with anti-human-specific Abs conjugated with FITC, PE, PEcy7, PerCP, PerCP-cy5.5, APC, or eFluor 660. These human Abs included anti–CD3, –CD4, –CD8, –CD45RA, –CD45RO, –CD62L, –CCR2, –CCR3, –CCR4, –CCR5, –CCR6, –CCR7, –IL-9, –IL-17, –IL-4, and –IFN-γ mAbs, which were purchased from BD Biosciences (Franklin Lakes, NJ), eBioscience (San Diego, CA), or R&D systems (Minneapolis, MN). Intracellular staining for IL-9, IL-17, IL-4, or IFN-γ was performed on T cells stimulated with phorbol myristate acetate (PMA, 50 ng/ml; Sigma-Aldrich St. Louis, MO) and ionomycin (1 µM; Sigma-Aldrich) in the presence of GolgiStop (BD Biosciences) for 5 h, and then stained with anti–IL-9, –IL-17, –IL-4, –IFN-γ, or –Foxp3 mAb conjugated with PE, PerCP-cy5.5, or PEcy7 (BD Biosciences or eBioscience). Intracellular staining for calretinin, a marker of mesothelial cells [34], was performed to identify PMCs. Fixed and permeabilized PMCs were primarily stained with mouse anti–human calretinin mAb (BD Biosciences), and then stained with FITC goat anti–mouse Igs (BD Biosciences). Appropriate species matched Abs served as isotype control. To explore the expression of molecules on PMCs, anti–IL-9R, –IL-4R, and –IFN-γR1 mAbs (R&D systems) conjugated with PE, PEcy7, PerCP or APC were used. Flow cytometry was performed on a FACS Canto II (BD Biosciences) and analyzed using BD FCSDiva Software and FCS Epress 4 software (De Novo Software, Los Angeles, CA).

### Cell isolation

Naïve CD4^+^ T cells were isolated from both TPE and blood by MACS based on negative selection using the Naïve CD4^+^ T cell isolation kit II (Miltenyi Biotec, Bergisch-Gladbach, Germany) according to the manufacturer's instructions. The purity of naïve CD4^+^CD45RA^+^ T cells was >97%, as measured by flow cytometry.

For isolating PMCs, the cell pellets of TPE were resuspended in RPMI-1640 (Gibco, Invitrogen, Carlsbad, CA) containing 20% heat-inactivated fetal bovine serum (FBS; Gibco), 20 ng/ml epidermal growth factor (R&D systems), and 50 µg/ml gentamycin. The cells were seeded into 25-cm^2^ flasks at a density of 1×l0^4^ cells/cm^2^ and placed in an incubator at 37°C in 5% CO_2_. After 24 h the monolayers were washed with HBSS to remove nonadherent cells and fresh media was added. The monolayers were monitored until confluent (7–10 d), then trypsinized, and subcultured for 5 to 6 passages. After each passage the cells grew to confluence within 4–5 d. In general, PMCs could be maintained for 6 to 7 passages before they became senescent.

### Differentiation of Th9 cells

Purified naïve CD4^+^ T cells from both TPE and blood (5×10^5^) were cultured in 1 ml of complete medium containing human IL-2 (2 ng/ml) in 48-well plates and stimulated with plate-bound anti-CD3 (OKT3; 1 µg/ml) and soluble anti-CD28 mAbs (1 µg/ml) for 7 d. The exogenous cytokines used were IL-1β (20 ng/ml), IL-4 (20 ng/ml), IL-6 (100 ng/ml), IL-12 (20 ng/ml), IL-21 (50 ng/ml), IL-25 (100 ng/ml), IFN-γ (30 ng/ml), and TGF-β (5 ng/ml). Recombinant human IL-1β, IL-4, IL-6, IL-12, IL-21, IL-25, IFN-γ and TGF-β were purchased from R&D Systems. In some experiments, designated numbers of PMCs were added into the culture.

### Th9 cell chemotaxis assays

Th9 cell *in vitro* chemotaxis assays were performed as previously described [33] with slight modifications. In briefly, the 8-µm pore polycarbonate filters in 24-well Transwell chambers (Corning Costar, Corning, NY) were used. Transwell membranes were coated with fibronectin (5 µg/ml; Chemicon International, Schwalbach, Germany) for 30 min at 37°C. Purified CD4^+^ T cells from blood (2×10^5^) were added into the top chamber resuspended in RPMI 1640 medium with 0.5% FBS in the final volume of 100 µl, TPE or supernatants of cultured PMCs were placed in the bottom chamber in a volume of 600 µl, and the chambers were incubated at 37°C in 5% CO_2_ atmosphere for 3 h. Finally, the total cells migrated into the bottom chamber were harvested and intracellular stained for IL-9 and then analyzed by flow cytometry as above described. The chemotaxis index was calculated by dividing Th9 cell numbers migrated in response to TPE or supernatants by Th9 cell numbers migrated in response to medium alone. To investigate whether CCL20 contributed to Th9 cell migration, blocking experiments were performed by mixing the TPE or supernatants with 100 ng/ml anti-CCL20 mAb or mouse IgG irrelevant isotype control (R&D systems).

### PMC in vitro injury model and long term restoring

Confluent monolayers of cultured PMCs were scratched with the tip of a p-200 pipette to create a uniform cell-free zone in each well. Cellular debris was removed by PBS washing. Wounded monolayers were then incubated in the presence of medium alone, IL-9 (100 ng/ml; R&D Systems), IL-4 (100 ng/ml; R&D Systems), or IFN-γ (30 ng/ml; R&D Systems). No epidermal growth factor was added into the culture. Microscopy pictures were taken at different time points after injury with a digital camera. The residual gap between migrating PMCs was measured with a computer-assisted image analysis system (Axiovision; Zeiss) and expressed as a percentage of the initial scratched area.

For long term restoring study, PMCs were cultured in the same conditions as the above wound healing experiments for up to 14 d, and then were photographed in the 0, 7, and 14 d and analyzed by calculating PMCs density using the same system.

### Apoptosis of PMCs *in vitro*

Confluent monolayers of PMCs were cultured in the presence of medium alone, IL-9 (100 ng/ml), IL-4 (100 ng/ml), IFN-γ (30 ng/ml) or their various combinations. After 48 h, PMCs were trypsinized and harvested, then stained with APC conjugated Annexin V and propidium iodide (Annexin V Apoptosis Detection Kit APC; eBioscience) and incubated at room temperature in dark for 10 min. Finally, the proportional apoptosis of PMCs was determined by flow cytometry.

### Antigen presentation to CD4^+^ T Cells by PMCs *in vitro*

Purified naïve CD4^+^ T cells (2×10^5^) from blood were cultured with autologous PMCs at a ratio of 5 ∶ 1 in RPMI-1640 supplemented with penicillin (100 U/ml), streptomycin (100 µg/ml), L-glutamine (2 mM), HEPES (10 mM), 10% type AB human serum in flat bottomed 96-well plates. In some experiments, IL-9 (100 ng/ml), IL-4 (100 ng/ml), IFN-γ (30 ng/ml), or ESAT-6/CFP-10 (10 µg/ml, State Key Laboratory of Agricultural Microbiology, Huazhong Agricultural University, Wuhan, China) were added into the culture. Suspension cells were harvested at 5 d, then Th9 cells within CD4^+^ T cells were determined by flow cytometry as above described.

In some experiments, anti-CD80 (10 µg/ml) or/and anti-CD86 (10 µg/ml) mAbs (eBioscience), or CTLA-4Ig (5 µg/ml; R&D systems) or control Igs (mouse IgG_1k_ (10 µg/ml) for anti-CD80 and anti-CD86 and mouse IgG_2a_ (5 µg/ml) for CTLA-4Ig) were added. To calculate percentage inhibition, basal unstimulated proliferation was subtracted and then percentage inhibition was calculated relative to its mAb control.

### Statistics

Data are expressed as mean ± SEM. Comparisons of the data between different groups were performed using a Kruskal-Wallis one-way analysis of variance on ranks. For variables in TPE and in corresponding blood, paired data comparisons were made using a Wilcoxon signed-rank test. The correlations between variables were determined by Spearman rank correlation coefficients. Analysis was completed with SPSS version 16.0 Statistical Software (Chicago, IL, USA), and p values of less than 0.05 were considered to indicate statistical significance.
